# OVERVIEW: THE VA GERIATRIC SCHOLARS PROGRAM FOR PRIMARY CARE WORKFORCE DEVELOPMENT

**DOI:** 10.1093/geroni/igz038.2814

**Published:** 2019-11-08

**Authors:** Josea Kramer, Joe Douglas, Shawn Clarke, Luis Melendez

**Affiliations:** 1 VA Greater Los Angeles Healthcare System GRECC, Los Angeles, California, United States; 2 GLA GRECC, Golden, Colorado, United States; 3 VA Greater Los Angeles Healthcare System GRECC, Sepulveda, California, United States; 4 VA Greater Los Angeles Healthcare System, Sepulveda, California, United States

## Abstract

The VA has invested in developing the skills of its primary care workforce through the longitudinal Geriatric Scholars Program. The program consists of core components --- intensive course in geriatrics, intensive workshop in quality improvement (QI) and initiation of a micro QI projects in the Scholar’s clinic; electives allow learners to tailor the program to self-identified gaps in knowledge, skills and competencies. The program has demonstrated direct impacts of continuing education through a workforce development process that enhances skills and competencies at a pace and selection that meets clinicians’ self-identified gaps in training. Now in its 11th year, the program has been shown to increase career satisfaction and job retention, standardize provider behaviors, improve clinical decision-making and reduce dispensing of potentially inappropriate medications. This symposium further explores the impact of the program on individual clinicians and on clinical teams.

